# Is Graph Theoretical Analysis a Useful Tool for Quantification of Connectivity Obtained by Means of EEG/MEG Techniques?

**DOI:** 10.3389/fncir.2018.00076

**Published:** 2018-09-26

**Authors:** Maciej Kaminski, Katarzyna J. Blinowska

**Affiliations:** ^1^Department of Biomedical Physics, Faculty of Physics, University of Warsaw, Warsaw, Poland; ^2^Institute of Biocybernetics and Biomedical Engineering of Polish Academy of Sciences, Warsaw, Poland

**Keywords:** graph theoretical analysis, effective connectivity, brain networks, directed transfer function, assortative mixing

Publication of Euler (1736) is regarded as the first paper on graph theoretical analysis (GTA), however, the term graph was introduced later by Sylvester (1878). Among the first applications of GTA were papers of Percheron (1982) on natural binary arborescences and of Kohn and Letzkus (1983) on metabolic regulation. GTA formalism assumes that a basic representation of a network—a graph—consists of nodes (vertices) and connections between them (edges). The application of GTA for quantification of connectivity structure in brain gained the impetus with a work of Watts and Strogatz (1998), who popularized the so-called “small world” (SW) networks. The basic parameters describing a network are clustering coefficient (CC) and path length (PL). CC is a measure of the local interconnectedness of a graph—a fraction of the neighbors of a node that are also neighbors of each other. PL is the shortest path between two vertices expressed as the number of traversed vertices. Every node of the network should be connected with every other node by at least one path. “Small-world” networks are characterized by a high CC and a short PL. These parameters are normalized in respect of reference parameters obtained from equivalent random networks. The network is considered to be “small world” if the ratio of the normalized CC to PL is >1. The other parameters frequently used to characterize networks are the node degree (the number of edges connected to a given vertex), the average number of edges for vertex and the global efficiency. The further developments in GTA theory included scale-free networks, a class of networks that as a whole had a power-law distribution of the number of links connecting to a node (Barabási and Albert, 1999). Local properties of networks can be described as motifs—recurrent and statistically significant sub-graphs or patterns (Shen-Orr et al., 2002). The network has community structure if the nodes can be easily grouped into sets such that each set of nodes is densely connected internally with a weaker connections between groups (Newman and Girvan, 2004). Network modularity reflects the concentration of edges within modules compared with a random distribution of edges between all nodes (Newman, 2003).

GTA finds applications in many branches of science in particular in brain studies. Recently a criticism of GTA and in particular of SW formalism application to quantify brain connectivity patterns was raised. Herein we shall concentrate on the application of GTA to the quantification of networks obtained by means of EEG/MEG and the pitfalls of this approach.

In the publications on GTA applications to brain signals, the “small world” topology was usually searched and the CC and PL parameters were used to distinguish between the experimental conditions or groups of subjects. However, results obtained for networks based on EEG/MEG signals by different researchers quite often yielded divergent or contradictory results. Compare the Alzheimer disease study with EEG and MEG techniques by the same group (Stam et al., 2007), (Stam et al., 2009). In studies of schizophrenia, a divergence of SW parameters determined by different researchers was reported; in some cases, the small world index did not distinguish between populations of healthy controls and schizophrenics at all (Rutter et al., 2013).

Inspecting works based on bivariate measures and graph theoretical analysis, it is difficult to find compatibility with imaging and electrophysiological evidence. A good example is the MEG finger movement experiment (Basset et al., 2006) in which wavelet analysis and correlations were followed by GTA. No statistically significant changes in GTA parameters depending on frequency were found between rest and movement and no lateralization was observed. It is a known fact that the movement task is connected with drastic changes in the topographic and spectral characteristics of brain activity (Pfurtscheller, 1999). The multivariate method called Directed Transfer Function (DTF) used in movement task yielded very distinct, frequency dependent connectivity patterns (Ginter et al., 2001; Kuś et al., 2006).

Also in the studies of sleep EEG in which connectivity was estimated using SL and quantified by GTA, no significant changes in the network parameters were found for different sleep stages (Ferri et al., 2005; Leistedt et al., 2009). Already in 1997, in the study in which the DTF multivariate method was used, very distinct connectivity patterns (frequency dependent and different for each sleep stage) were reported (Kaminski et al., 1997). The slow wave sleep EEG connectivity patterns obtained using SL and DTF are shown in Figures 1A,B respectively. In Figure 1A the network looks unclear, and in Figure 1B a propagation from the electrode overlying the corpus callosum (a structure which the neural tracts diverge from) was observed. Indeed, in slow wave sleep, the EEG activity is strongly synchronized, which can be attributed to driving from a common source, located in a place of divergence of nerves.

**Figure 1:**
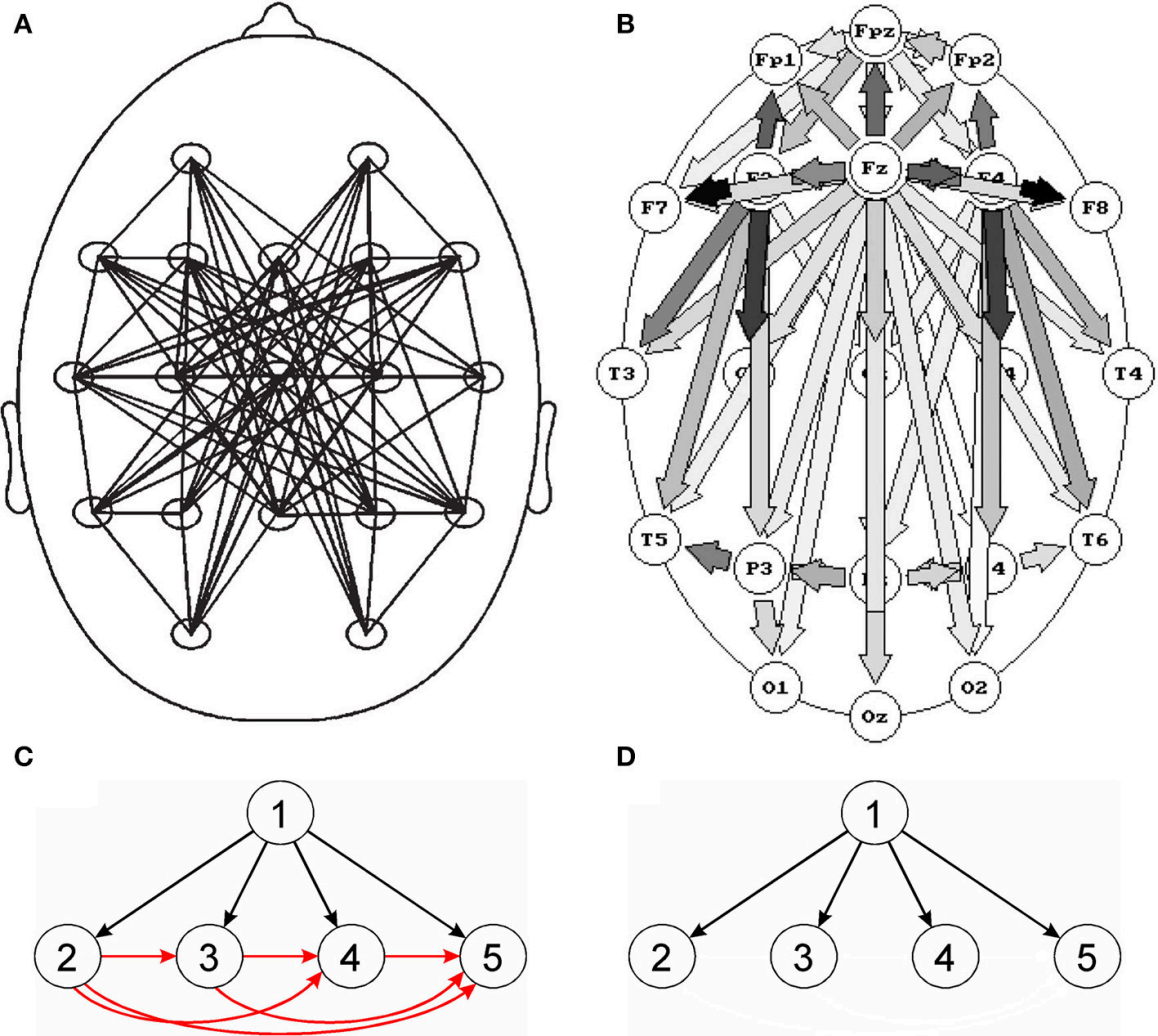
Comparison of bivariate and multivariate connectivity measures. Top images: connectivity patterns for slow wave sleep (stage 3/4), **(A)** obtained using the bivariate measure (SL), **(B)** obtained using the multivariate measure (DTF). Although in **(A)** undirected and in **(B)** directed connections are shown, however the main difference between the pictures are: disorganized pattern of connections in **(A)** and clear-cut pattern of connections compatible with physiological evidence in **(B)**. Bottom images—propagation patterns for a simulation which assumes a propagation of activity from electrode 1 to electrodes 2, 3, 4, and 5; **(C)**—pattern obtained for a bivariate measure (coherence) and **(D)**—for a multivariate measure (DTF). For the bivariate connectivity measure, false connections are created resulting from common driving. **(A)** Reproduced from Leistedt et al. (2009). **(B)** Reproduced from Kaminski et al. (1997) (with permission).

The accumulated criticism of GTA application in EEG/MEG studies mainly concerns the assessment of the connectivity structure. There are several factors which critically influence the results: (1) connectivity estimation method, (2) sensor density, (3) setting connection thresholds, (4) normalization method against random networks.

The first step of the analysis, the recording of signals, influences the network structure, particularly the number of nodes since recording sensors usually constitute network nodes. In the case of very dense distribution of sensors, they measure highly correlated activity, which may lead to an increase in the clustering coefficient.

The SW parameters are sensitive to thresholds used to eliminate non-significant connections. The GTA formalism requires that networks have no unconnected nodes. In order to meet that assumption, connection thresholds are quite often arbitrarily set to low values, (e.g., Stam et al., 2007; Van Heuvel et al., 2008; Leistedt et al., 2009), which increases the number of edges. Sometimes the threshold values were individually adapted in such a way that all considered networks had the same average number of edges per vertex (e.g., Leistedt et al., 2009). As the authors themselves admit, the GTA parameters are quite sensitive to the choice of thresholds (Stam et al., 2007). While for high threshold values networks seem to be organized into modules with large-world self-similar properties, the addition of a few weak links can make a network small-world. However, when the number of sensors is small, the overall network size is also small, PL does not vary much and the value of σ, characterizing “smallworldness” depends mainly on CC (Papo et al., 2016).

Another problem concerning GTA application is the reliability of SW structure identification against random networks. The random network construction procedure usually involves random rewiring of a network, but typically the number of nodes, links, and the degree distribution are not changed. In this way, some features of the investigated network are preserved in the reference random network. Hlinka et al. (2017) demonstrated that the model process with randomly scrambled interconnections reveals SW features similar to the ones of the original time series.

However, the most serious pitfall of the SW approach is the common drive effect. In particular, when bivariate methods of connectivity estimation are used, many spurious connections may be produced. The effect is illustrated in Figure 1C. If the source activity (channel 1) is recorded by channels 2, 3, and 4, bivariate measures will not only show connections between channel 1 and channels 2, 3, and 4 but also between all the channels which record activity from channel 1 owing to common driving. In effect, for *N* channels which record activity from a given source, we will obtain *N* true and *N*(*N* − 1)/2 false connections (Blinowska and Kaminski, 2013). When the number of sensors *N* measuring activity from given sources increases, the number of true connections increases linearly with *N* and the number of false connections increases as *N*^2^. This effect is responsible for multiple connections obtained using bivariate measures, e.g., Figure 1A. Setting the connection values above a given threshold to the same value, which is common practice, further blurs the connections pattern (Blinowska and Kaminski, 2013).

The common drive effect may be avoided by fitting all the channels into one model e.g., multivariate autoregressive model (MVAR)–Figure 1D. The methods derived from the MVAR model, DTF (Kaminski and Blinowska, 1991) or PDC—Partial Directed Coherence (Baccala and Sameshima, 2001), are free from the common drive effect. These estimators can be considered as extensions of the Granger causality concept (Kaminski et al., 2001), so they yield causal, directed connectivity.

When spurious connections caused by the common drive effect are eliminated, the connectivity patterns become very sparse and show clear-cut connectivity patterns. The relevant examples are: EEG sleep studies (Kaminski et al., 1997), finger movement experiment (Ginter et al., 2001; Kuś et al., 2006), Continuous Attention Test (Blinowska et al., 2010), and working memory task (Blinowska et al., 2013), which were compatible with the known imaging, electrophysiological and anatomical evidence.

In a working memory experiment involving memorizing and recollection of Greek letters by groups of less and better-educated people, EEG analysis was performed by calculation of SL followed by GTA (Micheloyannis et al., 2006). No characteristic frequency or topographic features were identified in the obtained connection patterns, except that for less educated people the SW connection pattern was reported and for better-educated people the connectivity structure was random. In a similar working memory task with by Kitzbichler et al. (2011) in which phase synchronization was considered, the dense connection pattern did not indicate brain regions involved in information processing.

A similar paradigm involving memorizing letters and relationships between them was applied in Blinowska et al. (2013) where effective connectivity was determined using time-varying DTF. A clear-cut picture of transmissions between the main centers of propagation located in the frontal and parietal regions was observed, which was in agreement with the imaging studies (Brzezicka et al., 2011) and the neurophysiological hypotheses concerning the mechanisms of WM (Acuna et al., 2002).

Neural networks obtained using multivariate methods are too sparse (disconnected) to apply the SW formalism, however, in order to assess the community structure of networks, more advanced methods such as assortative mixing (Newman and Girvan, 2004) may be applied. Using the above method, the existence of a modular structure of brain networks with a higher connection density within modules than between them was demonstrated (Blinowska et al., 2013). Moreover, dynamical analysis of information processing in the WM task showed frequency specific information transfer occurring transiently between distant neural populations.

The challenges to “smallworldness” were brought by recent publications based on tract-tracing. Kohn and Letzkus (1983); Bassett and Bullmore (2017) indicate that large-scale neuronal networks of the brain are arranged as globally sparse hierarchical modular networks.Bassett and Bullmore (2017) recommend an application of weighted graphs, which retain more information of biological relevance. They underline a role of strongly coupled clusters which comprise functionally specialized areas of cortex and importance of replicable, but weak connections between them. The approach applied by us (Blinowska et al., 2013) based on assortative mixing complies with the above-mentioned information on brain organization. The assortative mixing approach can be applied to sparse, weighted, and directed networks and allows for identification of the topography-specific modular structure of networks.

We can conjecture that the concept of “smallworldness” faced with the contemporary evidence based on experimental facts and methodological considerations is not adequate for quantification of EEG/MEG based networks structure. On one hand, there are several methodological pitfalls which may invalidate the results, on the other hand, SW concept based on dense connectivity structure produced by bivariate methods is not able to grasp the complicated nature of neural networks, in consequence, its information content is too low.

For connectivity assessment of EEG/MEG data, we recommend multivariate methods free from common feeding effect and yielding weighted and directed networks. For quantitative analysis of networks patterns, the assortative mixing is a good choice, since this method allows for identification of community network structure including topography specific modules and strengths of coupling within and between them. This kind of approach gives a rich topographical and functional information on brain organization.

## Author contributions

All authors listed have made a substantial, direct and intellectual contribution to the work, and approved it for publication.

### Conflict of interest statement

The authors declare that the research was conducted in the absence of any commercial or financial relationships that could be construed as a potential conflict of interest.
